
JOURNAL INFORMATION
==============================
NLM Title Abbreviation: Brain Commun
Journal ID: Brain Commun
NLM Journal ID: 101755125
Journal ID: braincomms

Brain Communications

EISSN: 2632-1297

ARTICLE INFORMATION
==============================
PMCID: PMC8500117
PMID: 34667948
DOI: 10.1093/braincomms/fcab219
Article ID: fcab219
Article version: 1
Subjects: Letter to the Editor, AcademicSubjects/MED00310, AcademicSubjects/SCI01870

Serum and cerebrospinal fluid biomarker profiles in acute SARS-Co-2 associated Guillain-Barré syndrome

Berciano José
Professor emeritus ad honorem, University of Cantabria, University Hospital “Marqués de Valdecilla (IDIVAL) , ” and “Centro de Investigación Biomédica en Red de Enfermedades Neurodegenerativas (CIBERNED), ”, Santander, Spain
Correspondence to: José Berciano, Professor emeritus ad honorem, University of Cantabria, Spain, E-mail: joseberciano51@hotmail.com
Electronic publication date: 2021 Sep 25
Electronic Location ID: fcab219
Received 2021 Aug 17; Accepted 2021 Aug 23
Copyright: © The Author(s) (2021). Published by Oxford University Press on behalf of the Guarantors of Brain.
Copyright year: 2021
License: This is an Open Access article distributed under the terms of the Creative Commons Attribution License (https://creativecommons.org/licenses/by/4.0/), which permits unrestricted reuse, distribution, and reproduction in any medium, provided the original work is properly cited.
License URL: https://creativecommons.org/licenses/by/4.0/

Keywords: COVID-19, endoneurial inflammatory oedema, endoneurial ischemia, Guillain-Barré, syndrome, NfL
Page count: 6
edited-state: accepted-manuscript
article-lifecycle: PAP

==============================

**Letter to the Editor**

### Serum and cerebrospinal fluid biomarker profiles in acute SARS-Co-2 associated Guillain-Barré syndrome

José Berciano

Professor emeritus *ad honorem*, University of Cantabria, University Hospital "Marqués de Valdecilla (IDIVAL)" and "Centro de Investigación Biomédica en Red de Enfermedades Neurodegenerativas (CIBERNED)", Santander, Spain

**Key words:** COVID-19; endoneurial inflammatory oedema; endoneurial ischemia; Guillain-Barré syndrome; NfL

**Correspondence to:** José Berciano, Professor emeritus *ad honorem*, University of Cantabria, Spain

E-mail: joseberciano51@hotmail.com

© The Author(s) (2021). Published by Oxford University Press on behalf of the Guarantors of Brain.
This is an Open Access article distributed under the terms of the Creative Commons Attribution License (http://creativecommons.org/licenses/by/4.0/), which permits unrestricted reuse, distribution, and reproduction in any medium, provided the original work is properly cited.

I read with great interest the paper by Paterson and colleagues evaluating serum and cerebrospinal fluid biomarkers in acute SARS-Covid-2-associated neurologic disorders including Guillain-Barré syndrome (GBS)[1]. Concerning GBS, they found that levels of neurofilament light protein (NfL) were elevated in serum, but normal in cerebrospinal fluid (CSF). Wisely the authors state that such finding implies that NfL is released from peripheral nerves into blood, and does not freely pass back into CSF. I wish to offer the pathological basis for this proposal.

In very early GBS (≤ 4 days after onset) the main pathogenic lesion is endoneurial inflammatory oedema predominating in nerve segments with less efficient blood-nerve barrier, including spinal nerves, spinal ganglia, spinal nerves, and pre-terminal and terminal nerve segments[2]. In nerve segments possessing epi-perineurium, namely external to subarachnoid angle and particularly in spinal nerves, oedema might be pathogenic by means of increasing endoneurial fluid pressure, which constricts transperineurial microcirculation producing rapid ischemic nerve injury, potential cause of distally accentuated Wallerian-like degeneration. Figure 1 illustrates drastic change of pathology between ventral spinal root and ventral ramus of spinal nerve at lumbar level[3]. Ultrasonographic studies have revealed that oedema of ventral rami of spinal nerves is a hotspot in early GBS, either demyelinating or axonal[4]. In severe GBS massive axonal damage in ventral rami of spinal nerves may be followed by retrograde axonal degeneration extending into ventral roots, eventually causing central chromatolysis on the anterior horn cells[2]. Needless to say, in acute motor axonal neuropathy Wallerian-like degeneration may also be accounted for by antiganglioside antibodies.

Assuming that the aforementioned increase of serum NfL was detected in early stages of GBS/COVID-19, I would suggest that it is correlated with axonal dysfunction caused by inflammatory oedema in extradural nerve trunks. Conceivably, in the course of the disease extension of axonal changes to spinal nerve roots could account for potential NfL changes in CSF.

**Data availability**

Data sharing is not applicable to this article as no new data were created or analysed.

**Competing interests**

The author reports no competing interests.

**References**

1. Paterson RW, Benjamin LA, Mehta PR, et al. Serum and cerebrospinal fluid biomarker profiles in acute SARS-CoV-2-associated neurological syndromes. Brain Commun. 2021; 3(3): fcab099. doi: 10.1093/braincomms/fcab099. eCollection 2021.
2. Berciano J. Axonal degeneration in Guillain-Barre syndrome: a reappraisal. J Neurol. 2020 Jun 30. doi: 10.1007/s00415-020-10034-y.
3. Berciano J, García A, Figols J, et al. Perineurium contributes to axonal damage in acute inflammatory demyelinating polyneuropathy. Neurology. 2000; 55(4): 552-559.
4. Gallardo E, Sedano MJ, Orizaola P, et al. Spinal nerve involvement in early Guillain-Barré syndrome: a clinico-electrophysiological, ultrasonographic and pathological study. Clin Neurophysiol. 2015; 126(4): 810-819.

### Figure legend

**Figure 1. Pathological findings in a fulminant GBS patient who died on day 60**

Electrophysiological studies (days 4, 17 and 50) initially showed normal nerve conduction velocities with further slowing, progressive attenuation of compound muscle action potentials, and profuse denervation. (**A**) The density of myelinated fibres appears preserved in this complete, semithin cross-section of L5 ventral root; note the absence of perineurium (x62 before reduction). (**B**) In this complete, cross-section of ventral ramus of L3 nerve there is widespread diminution of myelinated fibres, particularly in sub-perineurial regions. Note the presence of a wedge-shaped area exhibiting almost complete loss of myelinated fibres (arrows), a finding characteristic of endoneurial ischemia (x62 before reduction). Adapted from Berciano and colleagues[3].

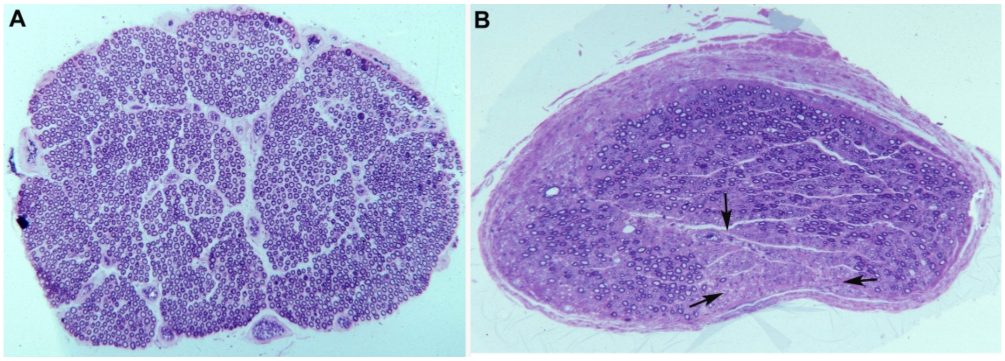

Figure 1. Pathological findings in a fulminant GBS patient died on day 60 (adapted from Berciano and colleagues3). Electrophysiological studies (days 4, 17 and 50) initially showed normal nerve conduction velocities with further slowing, progressive attenuation of compound muscle action potentials, and profuse denervation. (A) The density of myelinated fibres appears preserved in this complete, semithin cross-section of L5 ventral root; note the absence of perineurium (x62 before reduction). (B) In this complete, cross-section of ventral ramus of L3 nerve there is widespread diminution of myelinated fibres, particularly in sub-perineurial regions. Note the presence of a wedge-shaped area exhibiting almost complete loss of myelinated fibres (arrows), a finding characteristic of endoneurial ischemia (x62 before reduction).

720x254mm (300 x 300 DPI)
